# Interior Fracture Mechanism Analysis and Fatigue Life Prediction of Surface-Hardened Gear Steel under Axial Loading

**DOI:** 10.3390/ma9100843

**Published:** 2016-10-18

**Authors:** Wei Li, Hailong Deng, Pengfei Liu

**Affiliations:** School of Mechanical Engineering, Beijing Institute of Technology, Beijing 100081, China; 3120130209@bit.edu.cn (H.D.); liupengfei@bit.edu.cn (P.L.)

**Keywords:** surface-hardened steel, long life fatigue, interior fracture, inclusion, life prediction

## Abstract

The interior defect-induced fracture of surface-hardened metallic materials in the long life region has become a key issue on engineering design. In the present study, the axial loading test with fully reversed condition was performed to examine the fatigue property of a surface-carburized low alloy gear steel in the long life region. Results show that this steel represents the duplex *S-N* (stress-number of cycles) characteristics without conventional fatigue limit related to 10^7^ cycles. Fatigue cracks are all originated from the interior inclusions in the matrix region due to the inhabitation effect of carburized layer. The inclusion induced fracture with fisheye occurs in the short life region below 5 × 10^5^ cycles, whereas the inclusion induced fracture with fine granular area (FGA) and fisheye occurs in the long life region beyond 10^6^ cycles. The stress intensity factor range at the front of FGA can be regarded as the threshold value controlling stable growth of interior long crack. The evaluated maximum inclusion size in the effective damage volume of specimen is about 27.29 μm. Considering the size relationships between fisheye and FGA, and inclusion, the developed life prediction method involving crack growth can be acceptable on the basis of the good agreement between the predicted and experimental results.

## 1. Introduction

Because of the protective effect against surface fatigue fracture, the surface-hardening technology has been extensively employed to enhance the fatigue strength or life of structural materials such as low carbon alloy steels [1]. However, recent studies have shown that the surface-hardening sometimes cannot enhance fatigue performance of structural materials with respect to long life [2,3]. The reason is that there is a change of the fracture mode from the surface fatigue fracture at high stress level to the interior fatigue fracture at low stress level [3,4]. It is because of the transition of fatigue fracture mode or crack nucleation site that some surface-hardened or high-strength structural materials can present peculiar *S-N* characteristics [5,6] and complicated fracture mechanisms [7,8,9,10]. 

Surface fatigue fracture of structural materials is very common, which is often induced from irreversible cyclic slip or machining flaws. Moreover, the service environment can degrade the fatigue performance of structural materials and promote the occurrence of surface fatigue fracture. By contrast, interior fatigue fracture is mainly associated with some metallurgical defects of materials such as non-metallic inclusions or pores. For alloy steels, interior inclusion-induced crack nucleation and growth is the most common mode of interior fatigue fracture. Generally, a propagating crack shaped like a fisheye can occur on the fracture surface, and the inclusion is almost located at the center of the fisheye. Furthermore, it should be noted that a characteristic rough area with granular morphology sometimes can occur at the vicinity of the inclusion. This area is named as “fine granular area (FGA)” [5]. Some theories or ideas such as “depressive decohesion of spherical carbide” [6], “hydrogen embrittlement-assisted cracking” [7], “cyclic compression between crack faces” [8], “grain refinement and local stress decreasing” [9], and “numerous cyclic pressing and nanograin formation” [10] have been proposed to explain the formation mechanism of the FGA. Unfortunately, the uniform conclusion about the formation mechanism of the FGA has not yet been drawn. However, researchers all believe that the crack nucleation and growth behaviors within the FGA govern the fatigue properties of alloy steels with respect to long life.

Based on the idea that the crack nucleation is predominant in the long life fatigue process, several models related to dislocation theory [11] were developed to evaluate the crack nucleation life within the FGA, but it is still a difficult task since the crack nucleation mechanism within the FGA is not yet well understood. Conversely, studies [12,13] have shown that fatigue crack still can grow even though the stress intensity factor at the crack tip is below the traditional threshold value controlling long crack growth, and in vacuum the effective crack growth rate of some alloy steels can be reduced to 5 × 10^−13^ m/cycle [13]. Moreover, the crack morphology similar to the FGA is observed on the fracture surface of specimens during the crack growth rate testing in vacuum [9]. In view of the fact that the interior crack-induced fatigue fracture takes place in vacuum and the propagating crack can be formed in very few cycles [14], some researchers proposed [15,16] that fatigue life consumed in the FGA formation process could be evaluated from the viewpoint of crack growth.

In this study, the axial loading test was performed to experimentally examine the fatigue property of a surface-hardened gear steel in the long life region. Based on the analysis of *S-N* characteristics, fracture mechanisms, characteristic crack sizes, and severity of stress distribution around crack tip, a theoretical method of predicting the fatigue strength and life for the surface-hardened steel in its longevity was proposed. 

## 2. Experimental Procedure

### 2.1. Material and Specimen

**Figure 1:**
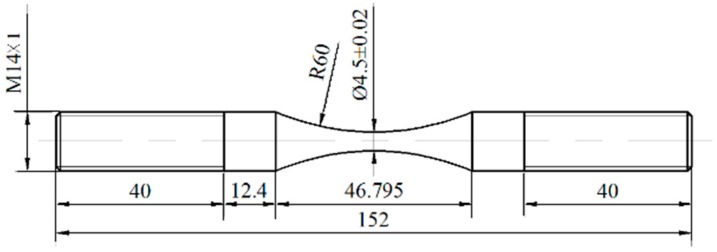
Shape and dimensions of specimen (units: mm).

The material investigated in this study is a low alloy Cr-Ni steel for drive gear, its main chemical composition (mass percentage) is 0.16 C, 0.37 Si, 0.60 Mn, 0.035 S, 0.035 P, 1.65 Cr, and 3.65 Ni. From the annealed steel bar with a diameter of 16 mm, first specimens were machined into the hourglass-shape with a certain amount of finishing margin, and then grinded in a direction of parallel to the axis of specimen by the grade 600–2000 abrasive paper to the final shape, as shown in Figure 1. The minimum diameter and the round-notched radius of the specimen are 4.5 mm and 60 mm, respectively. The corresponding elastic stress concentration factor, *K*_t_, is about 1.02 based on the book on “stress concentration factors” [17]. 

### 2.2. Carburizing and Microstructure

Specimens were placed into a container filled up with carburization powder that is composed of charcoal, calcium carbonate, and barium carbonate with 12:1:5. The container was sealed by fire clay and heated by a vacuum furnace. The furnace temperature first rises up to 800–850 °C, and then is held for 2 h or 4 h soaking time, finally reaching the carburizing temperature of about 930 °C. According to the speed of pack carburizing—about 0.1–0.15 mm/h—the carburizing time is about 8 h and the expected depth of carburized layer is about 0.8–1.2 mm. Upon completion of the process, the furnace temperature is stepped down to 850 °C for 30 min prior to quenching in oil, followed by tempering at 170 °C for 3 h. 

**Figure 2:**
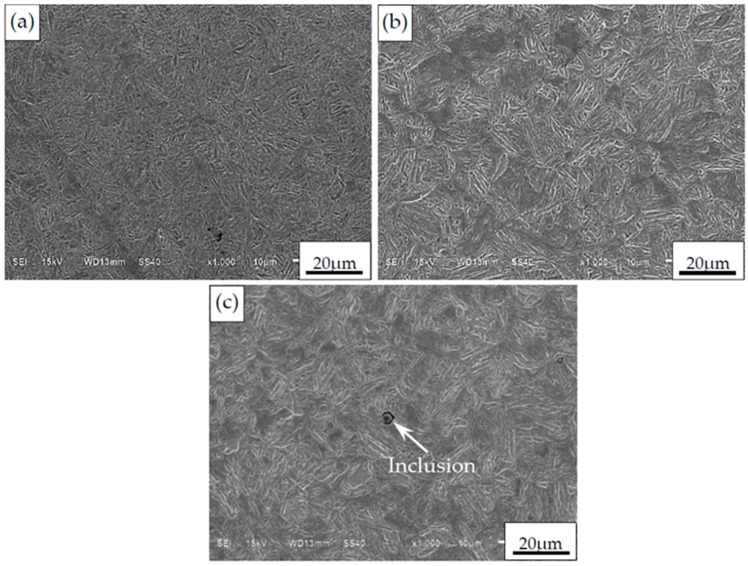
Observation of microstructure and inclusion. (**a**) Microstructure in carburized layer; (**b**) Microstructure in core region; (**c**) Inclusion.

After grinding and polishing, then etching with 4% alcohol nitric acid solution, the cross-sectional microstructure of specimen was observed by using the scanning electron microscopy (SEM). The microstructure in carburized layer differs from that in the core region, and the relevant morphologies are shown in Figure 2a,b, respectively. Combined with the analysis of energy dispersive X-ray spectrometer (EDS), the acicular martensites with high carbon and partial residual austenites can be observed in the carburized layer, whereas the lath martensites with low carbon can be observed in the core region. Moreover, some non-metallic inclusions of Al_2_O_3_ can be found in the microstructure, as shown in Figure 2c. 

### 2.3. Experimental Method

An electromagnetic resonant testing machine (CCQB Testing Co. Ltd., Changchun, China) at frequency of 100 Hz was used to perform the fatigue test of carburized Cr-Ni gear steel under axial loading. Fatigue testing at the constant stress ratio of −1 was performed at room temperature in an open environment. After the experiment, all the fracture surfaces of fractured specimens were carefully observed by the SEM. 

## 3. Results and Discussion

### 3.1. Micro-Hardness and Residual Stress

**Figure 3:**
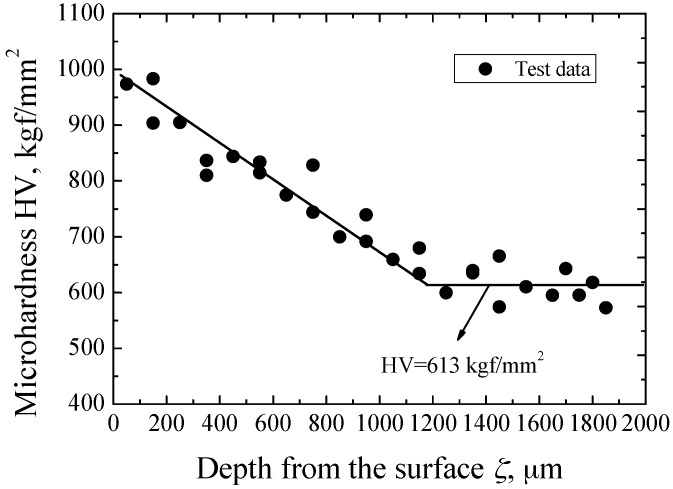
Distribution of micro-hardness.

By using an instrumented nano-indenter G200 with the measuring function of continuous stiffness, the micro-hardness on cross-sections of specimens was measured. Its distribution is characterized as a function of the depth from surface, *ζ*, as shown in Figure 3. At the near surface, the value of micro-hardness is the largest, and then tends to decrease. At the *ζ*-value of no less than about 1200 μm, it reaches a constant value of 613 kgf/mm^2^ that is the micro-hardness of untreated material. Thus, it can be concluded that the depth of the carburized layer is about 1.2 mm. 

Based on the sin^2^*ψ* method with Cr-K*α* radiation, the value of residual stress on the round-notch surface of specimen was measured along the axis of specimen by the TEC 4000 X-ray diffraction system. During testing, the tube voltage is 30 kV and the tube current is 6.7 mA. The maximum compressive residual stress occurs on the surface and is about 268 MPa by averaging the values of four measurement points on the surface.

### 3.2. S-N Characteristics

**Figure 4:**
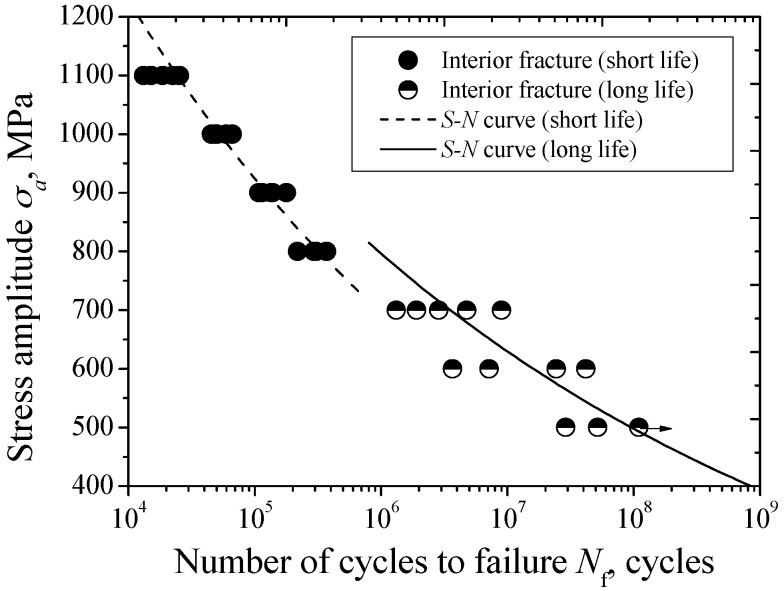
*S-N* diagram of carburized Cr-Ni gear steel under axial loading.

The *S-N* diagram of carburized Cr-Ni gear steel under axial loading is shown in Figure 4. Based on the preliminary SEM observation of fracture surface, especially crack nucleation site, the interior fatigue fracture becomes the predominant fracture mode in the life region of 10^4^–10^8^ cycles. Overall, this carburized steel presents the continuously descending *S-N* characteristics. The conventional fatigue limit corresponding to 10^7^ cycles cannot be found. 

Furthermore, these test data can be divided into two parts based on their distribution characteristics. One part corresponds to the short life region below 5 × 10^5^ cycles, while the other part corresponds to the long life region beyond 10^6^ cycles. Basically, the separation of these two parts of test data is distinct. Therefore, the duplex *S-N* curves are used to represent the fatigue *S-N* characteristics of carburized Cr-Ni gear steel under axial loading. In view of the nonlinear distribution feature of each part of test data on semilog coordinates, a Basquin model is used to establish these two *S-N* curves corresponding to the test data with short life and long life, respectively, plotted by a dashed line and a solid line in Figure 4.

### 3.3. Fatigue Fracture Mechanism

**Figure 5:**
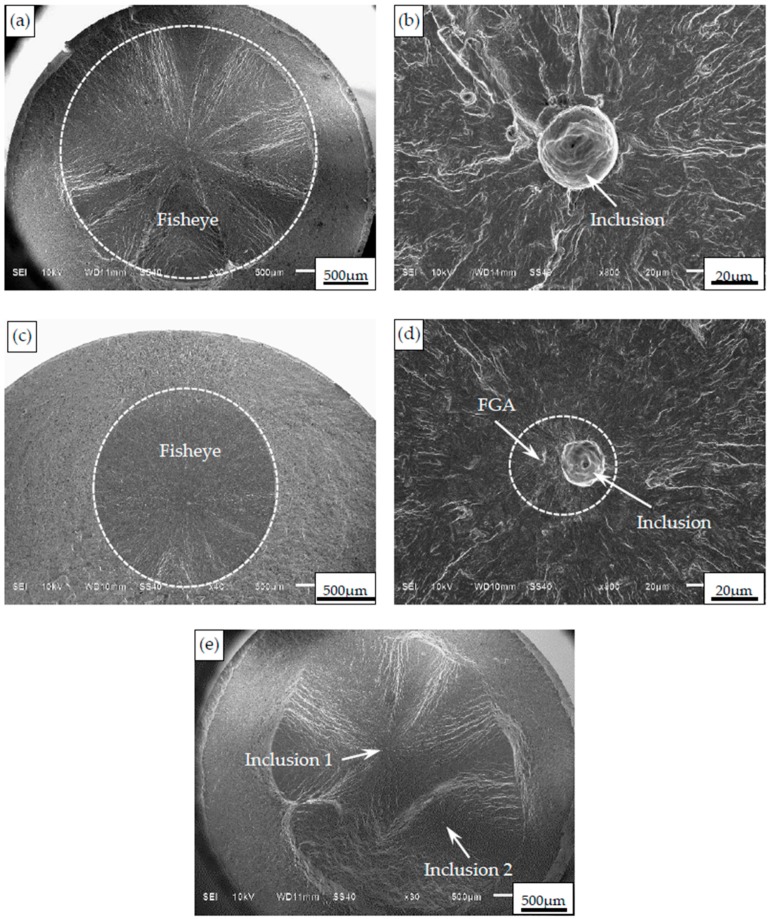
Observation of typical fracture surfaces. (**a**) Fisheye (*σ_a_* = 900 MPa, *N*_f_ = 114,800 cycles); (**b**) Inclusion without FGA (*σ_a_* = 900 MPa, *N*_f_ = 114,800 cycles); (**c**) Fisheye (*σ_a_* = 600 MPa, *N*_f_ = 41,727,000 cycles); (**d**) Inclusion with FGA (*σ_a_* = 600 MPa, *N*_f_ = 41,727,000 cycles); (**e**) Interference of two fisheyes (*σ_a_* = 1000 MPa, *N*_f_ = 45,500 cycles).

Based on the SEM observation and the EDS analysis, the interior fracture of specimens is all induced from nonmetallic inclusions in both the short and long timeframes. The fisheye can be observed on the fracture surface, as shown in Figure 5a,c. The inclusion is nearly located at the center of the fisheye. It should be noted that these inclusions are basically confined to the interior matrix region. In other words, the inclusions existing in the carburized layer hardly become crack nuclei due to the effect of the carburized layer. This means that the effective damage zone of a specimen under axial loading is just the interior matrix region.

In the short life region below 5 × 10^5^ cycles, the FGA cannot be observed at the vicinity of the inclusion, as shown in Figure 5b. Conversely, in the long life region beyond 10^6^ cycles, the FGA is observable at the vicinity of the inclusion, as shown in Figure 5c. This is consistent with the other experimental results of alloy steels with interior inclusion-induced fracture in the long life region [5,10,11]. Therefore, it can be confirmed that the formation of FGA is greatly related to the definite fatigue life. Approximately, the number of loading cycles with about 10^6^ is the lowest critical fatigue life for the FGA formation. 

In addition, the multiple fisheyes—i.e., multiple interior crack nucleation sites—can be observed on a fracture surface under high stress levels. Figure 5e shows the fracture surface morphology with two fisheyes. For these two fisheyes, fatigue cracks are all originated from the inclusions. With the increasing of loading cycles, two propagating cracks derived from two inclusions can interfere with each other. The interfered cracks can grow along the tangential direction of two fisheyes. In this case, it is concluded that the combined effects of two fisheyes should be responsible for the interior fatigue fracture of this specimen. Therefore, it is summarized that with the decrease of applied stress, the fatigue fracture of carburized Cr-Ni gear steel under axial loading successively represents the multiple inclusion-fisheye induced fracture, the single inclusion-fisheye induced fracture, and the single inclusion-FGA-fisheye induced fracture. 

### 3.4. Crack Size Characteristics

**Figure 6:**
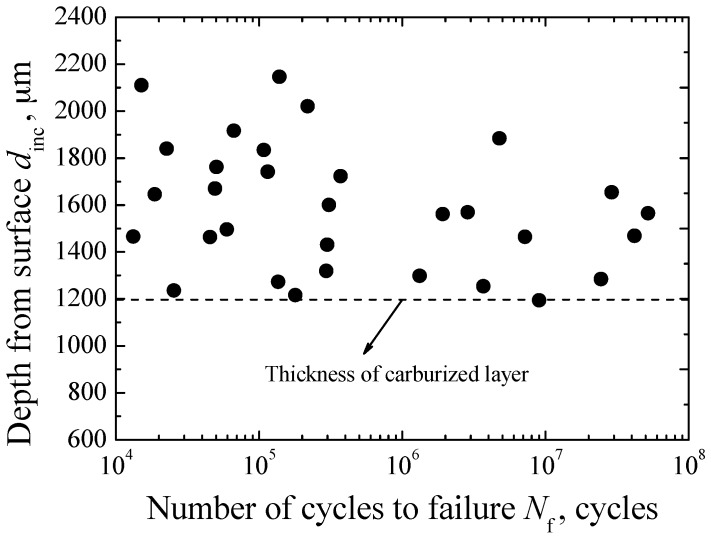
Relationship between *d*_inc_ and *N*_f_.

Based on the fractography, several crack size parameters were defined to discuss the interior fracture mechanism. First, the parameter *d*_inc_ denotes the depth of inclusion from its center to the nearest edge of fracture surface. By using Imagej software, the measured values of *d*_inc_ are in the range of 1216.11–2220.66 μm, as shown in Figure 6. They are almost all larger than the thickness of carburized layer. This further verifies the result of fracture surface observation. That is, the carburized layer can effectively inhibit the crack nucleation from the inclusions contained in itself. Furthermore, it can be seen that the values of *d*_inc_ are almost regardless of fatigue life. It is known that, under the axial loading, the stress distribution on the cross-section of specimen is uniform. Thus, combining the results of *d*_inc_, it is concluded that fatigue strength or life should be mainly associated with the sizes of inclusions in the matrix region, instead of the location of inclusions. 

**Figure 7:**
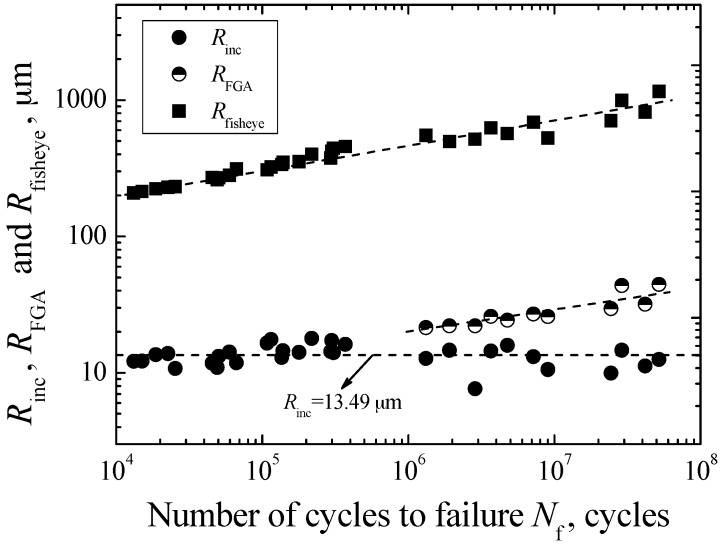
Relationships between *R*_inc_, *R*_FGA_, and *R*_fisheye_, and *N*_f_.

**Figure 8:**
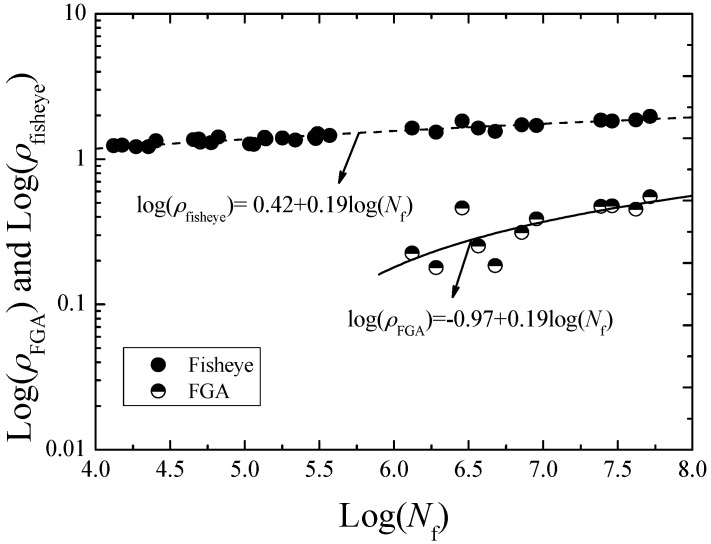
Relationships between *ρ*_fisheye_ and *ρ*_FGA_, and *N*_f_.

On the other hand, in view of the approximately circular shape of the inclusion, FGA, and fisheye, the parameters *R*_inc_, *R*_FGA_, and *R*_fisheye_ are used to indicate the radiuses of the inclusion, FGA and fisheye, respectively. The measured values of *R*_inc_, *R*_FGA_ and *R*_fisheye_ are shown in Figure 7. It can be seen that the values of *R*_inc_ are independent on fatigue life. The average value of *R*_inc_ is evaluated as 13.49 μm. However, for the values of *R*_FGA_ and *R*_fisheye_, they all tend to increase with the increasing of fatigue life. The parameters *ρ*_fisheye_ and *ρ*_FGA_ were defined to indicate the ratios of *R*_fisheye_ to *R*_inc_ and *R*_FGA_ to *R*_inc_, respectively. Figure 8 shows the relationships between *ρ*_fisheye_ and *ρ*_FGA_, and *N*_f_. It can be found that the values of *ρ*_fisheye_ and *ρ*_FGA_ all tend to increase with the increasing fatigue life, respectively indicated by a dashed curve and a solid curve in Figure 8. The corresponding curve equations are given by:

For fisheye:
  (1) $$\mathrm{Log}\left(\rho_{\mathrm{fisheye}}\right)=\mathrm{Log}\left(\frac{R_{\mathrm{fisheye}}}{R_{\mathrm{inc}}}\right)=0.42+0.19\mathrm{Log}\left(N_{f}\right)$$
For FGA:
  (2) $$\mathrm{Log}\left(\rho_{\mathrm{FGA}}\right)=\mathrm{Log}\left(\frac{R_{\mathrm{FGA}}}{R_{\mathrm{inc}}}\right)=-0.97+0.19\mathrm{Log}\left(N_{f}\right)$$

### 3.5. Evaluation of Interior Crack Growth

Studies [18,19,20] have shown that smaller inclusions or defects can be viewed as small cracks, and the propagating crack within the fisheye can be considered to have a circular shape. Therefore, under the stress ratio of −1, the stress intensity factor (SIF) range for an interior circular crack having a radius *a*, Δ*K*, can be determined by [20]:

(3) $$\Delta K=\frac{2}{\pi }\sigma_{a}\sqrt{\pi a}$$

In view of the fact that the tension has a predominant effect on the crack growth behavior but the compression has no significant effect, the value of Δ*K* in Equation (3) is evaluated by using the stress amplitude instead of the stress range. Moreover, Equation (3) is used with high accuracy even for a circular crack that is not concentric with the axis of the cylindrical specimen and has a radius up to one-half of the radius of the specimen [20]. Thus, the corresponding SIF values for the inclusion, FGA, and fisheye—Δ*K*_inc_, Δ*K*_FGA_, and Δ*K*_fisheye_—can be expressed as:

(4) $$\Delta K_{\mathrm{inc},\;\mathrm{or}\;\mathrm{FGA}\;\mathrm{and}\;\mathrm{or}\;\mathrm{fisheye}}=\frac{2}{\pi }\sigma_{a}\sqrt{\pi R_{\mathrm{inc},\;\mathrm{or}\;\mathrm{FGA}\;\mathrm{and}\;\mathrm{or}\;\mathrm{fisheye}}}$$

**Figure 9:**
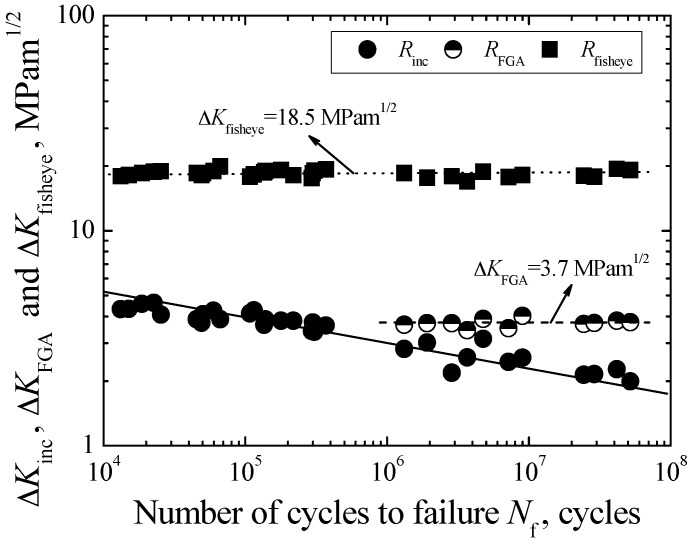
Relationships between Δ*K*_inc_, Δ*K*_FGA_, and Δ*K*_fisheye_, and *N*_f_.

Based on Equation (4), the evaluated values of ∆*K*_inc_ are in the range of 1.99–4.62 MPam^1/2^ and tend to increase with the increasing of *N*_f_, as indicated by a solid line in Figure 9. Like *S-N* data, the values of ∆*K*_inc_ also can be divided into two parts based on the difference of fracture mode. One part corresponds to the inclusion-fisheye induced fracture in the short life region below 5 × 10^5^ cycles, and the relevant values of ∆*K*_inc_ are in the range of 3.41–4.62 MPam^1/2^. Another part corresponds to the inclusion-FGA-fisheye induced fracture in the long life region beyond 10^6^ cycles, and the relevant values of ∆*K*_inc_ are smaller and only in the range of 1.99–3.14 MPam^1/2^. Moreover, Figure 9 shows the relationship between ∆*K*_FGA_ and *N*_f_. The values of Δ*K*_FGA_ are scattered in a limited range of 3.52–4.01 MPam^1/2^ with an average value of 3.7 MPam^1/2^, regardless of fatigue life. It is noted that the values of Δ*K*_FGA_ are similar to the partial values of ∆*K*_inc_ for the inclusion-fisheye induced fracture, and also similar to the threshold stress intensity factor governing long crack growth for some alloy steels, about 4 MPam^1/2^ [5,10,21]. 

**Figure 10:**
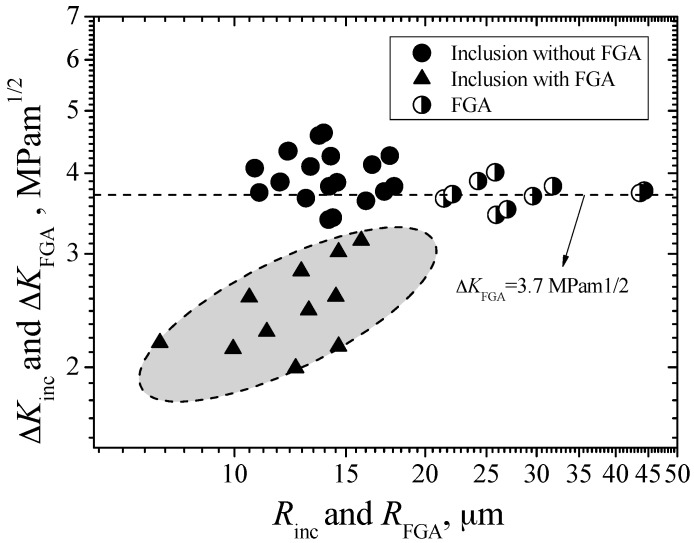
Relationships between Δ*K*_inc_ and Δ*K*_FGA_, and inclusion/FGA sizes.

Furthermore, the relationships between Δ*K*_inc_ and Δ*K*_FGA_, and inclusion/FGA sizes are established in Figure 10. It is clear that most of the values of Δ*K*_inc_ for the inclusion-fisheye induced fracture and most of the values of Δ*K*_FGA_ are all regardless of respective inclusion or FGA sizes. However, the values of Δ*K*_inc_ for the inclusion-FGA-fisheye induced fracture, shown in the shadow region of Figure 10, obviously tend to increase with the increasing of inclusion sizes. Therefore, Δ*K*_FGA_ can approximately be viewed as the threshold value controlling stable growth of interior long crack. This means that even if the inclusion belongs to the category of small crack in size, once its size exceeds the critical size corresponding to Δ*K*_FGA_ under a given stress level, the crack induced from this inclusion can directly enter the stable long crack growth stage. Conversely, if the inclusion size is less than the critical size corresponding to Δ*K*_FGA_ under a given stress level, the small crack growth can play a key role in the process of interior fracture. The threshold value controlling small crack growth from the inclusion is not a constant, but greatly proportional to inclusion size. 

In addition, Figure 9 also shows the relationship between ∆*K*_fisheye_ and *N*_f_. The values of ∆*K*_fisheye_ are also scattered in a limited range of 17.52–19.92 MPam^1/2^ with an average value of 18.5 MPam^1/2^, regardless of fatigue life. They are similar to the fatigue fracture roughness of steel. Therefore, ∆*K*_fisheye_ can be regarded as the threshold value for controlling unstable long crack growth. 

Therefore, from the viewpoint of crack growth, the interior fatigue fracture progress of carburized Cr-Ni gear steel in the long life region beyond 10^6^ cycles can be divided into three stages: (A) the small crack growth from the small inclusion to the FGA, controlled by ∆*K*_inc_; (B) the stable long crack growth from the FGA to the fisheye, controlled by Δ*K*_FGA_, or the stable long crack growth from the large inclusion to the fisheye, controlled by ∆*K*_inc_; (C) the unstable crack growth from the fisheye to the final fracture, controlled by ∆*K*_fisheye_. Among them, stage (C) is extremely short and can be negligible in the total life, whereas stage (A) determines the fatigue property in the long life region beyond 10^6^ cycles.

Firstly, studies [15,16] have shown that for the small crack growth in stage (A), the corresponding crack growth rate, *da*/*dN*, can be described by Paris equation, and given by:

(5) $$da/dN=C_{A}{\left(\Delta K\right)}^{m_{A}}$$

where *C*_A_ and *m*_A_ are the material-independent constants in this stage. Integrating of Equation (5) from the inclusion size, *R*_inc_, to the FGA size, *R*_FGA_, and combining with Equation (4), gives:

(6) $${\left(\frac{2}{\pi }\sigma_{a}\sqrt{\pi R_{\mathrm{inc}}}\right)}^{m_{A}}\left(\frac{N_{A}}{R_{\mathrm{inc}}}\right)=\frac{2}{C_{A}\left(m_{A}-2\right)}\left[1-{\left(\frac{R_{\mathrm{inc}}}{R_{\mathrm{FGA}}}\right)}^{\frac{m_{A}}{2}-1}\right]$$

Since more than 90% of total fatigue life is consumed in stage (A) [5,6], so fatigue life *N*_A_ can be approximatly equivalent to the total fatigue life *N*_f_. Combined with the measured sizes of inclusion and FGA, as well as the *S-N* data in the long life region beyond 10^6^ cycles, the values of *C*_A_ and *m*_A_ can be evaluated as 1.54 × 10^−15^ and 6.42, respectivley. Therefore, the interior crack growth rate in stage (A) is obtained as:

(7) $$da/dN=1.54\times 10^{-15}{\;(\Delta K)}^{6.42}$$

Next, for the stable long crack growth in stage (B), the corresponding crack growth rate also can be given by:

(8) $$da/dN=C_{B}{\left(\Delta K\right)}^{m_{B}}$$

where *C*_B_ and *m*_B_ are the material-independent constants in this stage. Because the fatigue life consumed in the process from the FGA to the fisheye cannot be determined, so the crack growth rate in this stage can be approximately evaluated by using the crack growth behavior from the large inclusion to the fisheye in the short life region. Integrating of Equation (8) from the inclusion size, *R*_inc_, to the fisheye size, *R*_fisheye_, and combining with Equation (4), Equation (8) can be rewritten as:

(9) $${\left(\frac{2}{\pi }\sigma_{a}\sqrt{\pi R_{\mathrm{inc}}}\right)}^{m_{B}}\left(\frac{N_{B}}{R_{\mathrm{inc}}}\right)=\frac{2}{C_{B}\left(m_{B}-2\right)}\left[1-{\left(\frac{R_{\mathrm{inc}}}{R_{\mathrm{fisheye}}}\right)}^{\frac{m_{B}}{2}-1}\right]$$

Based on the mearsued sizes of inclusion and fisheye, as well as the *S-N* data in the short life region below 5 × 10^5^ cycles, the values of *C*_B_ and *m*_B_ can be evaluatd as 1.21 × 10^−15^ and 7.72, respectively. Therefore, the interior crack growth rate in stage (B) is obtained as:

(10) $$da/dN=1.21\times 10^{-15}{\;(\Delta K)}^{7.72}$$

### 3.6. Evaluation of Maximum Inclusion and FGA Sizes

The maximum sizes of inclusion and FGA in a given volume of steel, *V*, were evaluated by using the statistics of extreme values (SEV) method [18]. Taking the inclusion as an example, firstly the inclusions observed from fracture surfaces are viewed as the maximum inclusions in a given set of inspection planes, *S*_0_. Herein, *S*_0_ is defined as the effective damage zone of minimum cross-section of specimen under axial loading—i.e., the interior matrix region, about 3.46 mm^2^. 

Then, let *X* be the dimension of extreme inclusions, and the inclusion sizes are assumed to be well characterized by the Gumbel distribution function, *F*(*x*), given by:

(11) F(x)=exp{−exp{−[(x−λ)/α]}}

where *λ* and *α* are location parameter and scale parameter, respectively. The size of *i*th inclusion, *x_i_*, are classified, starting from the smallest and indexed with *i* = 1, 2, ···, *n*, where *n* denotes the number of inclusion. For the small sample, the cumulative probability corresponding to *x_i_* is given by:

(12) $$P(x_{i})=(i-0.3)/(n+0.4)$$

According to Equations (11) and (12), the following equations are obtained as:

(13) $$x_{i}=\alpha y_{i}+\lambda$$

(14) $$y_{i}=-\ln\{-\ln[\left(i-3\right)/(n+0.4)]\}$$

**Figure 11:**
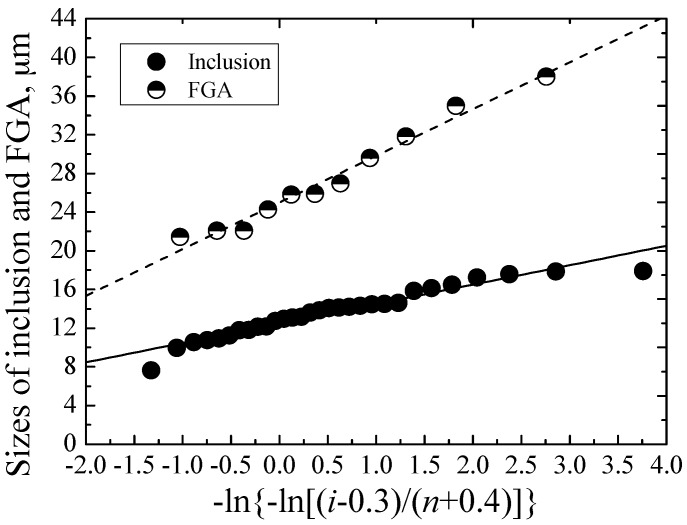
Evaluation of slope *α* and intercept *λ*.

The relationship between *x_i_* and *y_i_* is shown in Figure 11, which is described by a solid line with slope *α* and intercept *λ*. Based on the least square method, the values of *λ* and *α* are evaluated as 12.51 and 2.01, respectively. 

Next, the maximum inclusion is expected to be exceeded once in this volume. Let *X_V_* denote the characteristic value of maximum inclusion size, so the return period, *T*, and the cumulative probability of *X_V_*, *P*(*X_V_*), are given by:

(15) $$T=V/V_{0}$$

(16) $$P(X_{V})=1-1/T$$

where *V*_0_ is the volume of inspection plane with a certain thickness. The thickness is defined as the mean inclusion size, about 13.49 μm, so the value of *V*_0_ is evaluated as about 0.047 mm^3^. Therefore, the value of *X_V_* for the inclusion can be expressed as:

(17) $$X_{V}=12.51-2.01\ln[-\ln(1-0.047/V)]$$

Similarly, for the FGA, the relationship between *x_i_* and *y_i_* is indicated by a dashed line in Figure 11, and the values of *λ* and *α* are evaluated as 25.01 and 4.83, respectively. The mean size of FGAs is about 27.45 μm, so the value of *V*_0_ for the FGA is evaluated as about 0.095 mm^3^. Thus, the value of *X_V_* for the FGA is given by:

(18) $$X_{V}=25.01-4.83\ln[-\ln(1-0.095/V)]$$

**Figure 12:**
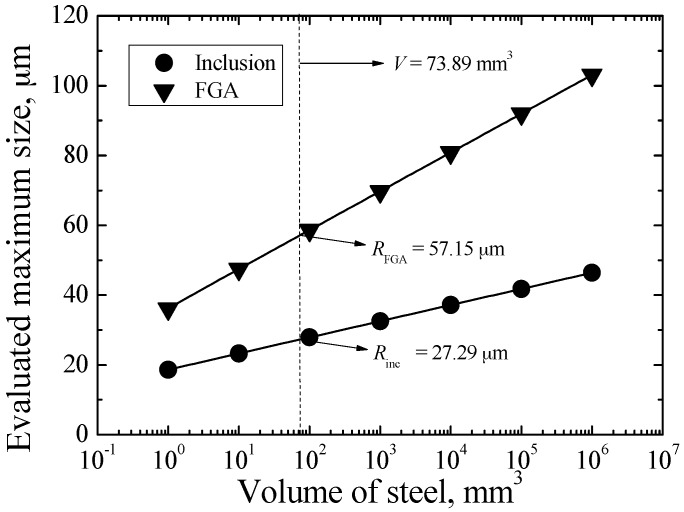
Evaluation of maximum sizes of inclusion and FGA.

Figure 12 shows the evaluated maximum sizes of inclusion and FGA as a function of *V* for carburized Cr-Ni gear steel. Apparently, they all tend to increase with the increasing of *V*. For the specimen under axial loading, the tested volume *V* can be expressed as [22]:

(19) $$V=0.25\pi lq^{2}$$

where *q* is the diameter of matrix region of minimum cross-section, *l* is defined as the length at which the stress value on the cross-section is 0.9 times that on minimum cross-section at *l*/2. For the tested specimen in this study, the values of *q* and *l* are 2.1 mm and 21.3 mm, respectively. Based on Equation (19), the value of *V* is evaluated as about 73.89 mm^3^. Then, based on Equations (17) and (18), the maximum inclusion and FGA sizes for the specimen in this study are evaluated as about 27.29 μm and 57.15 μm, respectively.

### 3.7. Prediction of Fatigue Life

Combined with Equations (2) and (6), fatigue strength for the inclusion-FGA-fisheye induced fracture in the long timeframe can be given by:

(20) $$\sigma_{a}={\left(\frac{2}{C_{A}\left(m_{A}-2\right)}\left[1-{\left(10^{-0.97+0.19\mathrm{Log}\left(N_{f}\right)}\right)}^{\frac{m_{A}}{2}-1}\right]\right)}^{1/m_{A}}\frac{\sqrt{\pi }}{2}R_{\mathrm{inc}}^{\left(2-m_{A}\right)/2m_{A}}N_{f}^{-1/m_{A}}$$

Combined with Equations (1) and (9), fatigue strength for the inclusion-fisheye induced fracture in a short timeframe can be given by:

(21) $$\sigma_{a}={\left(\frac{2}{C_{B}\left(m_{B}-2\right)}\left[1-{\left(10^{0.42+0.19\mathrm{Log}\left(N_{f}\right)}\right)}^{\frac{m_{B}}{2}-1}\right]\right)}^{1/m_{B}}\frac{\sqrt{\pi }}{2}R_{\mathrm{inc}}^{\left(2-m_{B}\right)/2m_{B}}N_{f}^{-1/m_{B}}$$

**Figure 13:**
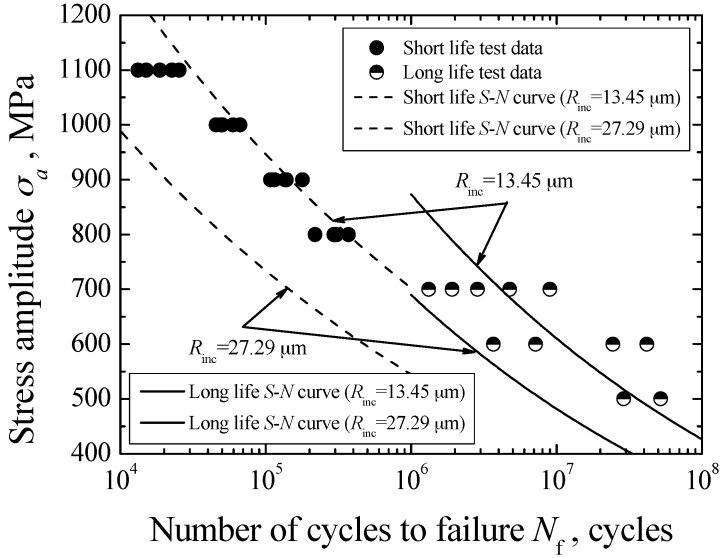
Prediction of fatigue life.

Therefore, only the values of *C*_A_ and *m*_A_ or the values of *C*_B_ and *m*_B_, as well as the inclusion size, are known, the interior *S-N* curve corresponding to different fracture mechanisms can be established by using Equations (20) and (21). Based on the mean and maximum inclusion sizes, the predicted interior *S-N* curves of carburized Cr-Ni gear steel are plotted in Figure 13. The predicted results using the mean inclusion size are in good agreement with experimental data while, by using the maximum inclusion size, effectively indicate the lower boundary of experimental data. In general, the life prediction method involving crack growth for the inclusion-FGA-fisheye induced fracture and the inclusion-fisheye induced fracture can be acceptable. It is noted that the method proposed in this study is mainly based on fatigue fracture mechanisms consisting of inclusion-fisheye induced fracture and inclusion-FGA-fisheye induced fracture. Only if the materials exhibit these fatigue fracture mechanisms, this method can be used to evaluate the relevant fatigue life or strength. Certainly, the applicability of this method still needs to be verified by the experimental results of other materials. 

## 4. Conclusions

Main conclusions obtained in this study are summarized as follows:

(1) The carburized Cr-Ni gear steel exhibits the constantly decreasing duplex *S-N* property without the traditional fatigue limit related to 10^7^ cycles.
(2) Fatigue cracks are all originated from the inclusions limited in the matrix region due to the inhabitation effect of the carburized layer.
(3) The inclusion-fisheye induced fracture is the main fracture mechanism in the short timeframe, whereas the inclusion-FGA-fisheye induced fracture is the main fracture mechanism in the long timeframe.
(4) Δ*K*_FGA_ can be viewed as the threshold value controlling stable long crack growth, and ∆*K*_fisheye_ can be regarded as the threshold value for controlling unstable long crack growth.
(5) The evaluated maximum sizes of inclusion and FGA in the effective damage volume of specimen are 27.29 μm and 57.15 μm, respectively.
(6) The proposed life prediction method involving crack growth can be acceptable based on the good agreement between predicted and experimental results.

## References

[1] Akita M., Tokaji K. (2006). Effect of carburizing on notch fatigue behavior in AJSI 316 austenitic stainless steel. Surf. Coat. Technol..

[2] Shiozawa K., Lu L. (2002). Very high-cycle fatigue behavior of shot-peened high-carbon-chromium bearing steel. Fatigue Fract. Eng. Mater. Struct..

[3] Shiozawa K., Murai M., Shimatani Y., Yoshimoto T. (2010). Transition of fatigue failure mode of Ni-Cr-Mo low-alloy steel in very high cycle regime. Int. J. Fatigue.

[4] Naito T., Ueda H., Kikuchui M. (1984). Fatigue behavior of carburized steel with internal oxides and nonmartensitic microstructure near the surface. Metall. Trans..

[5] Sakai T., Sato Y., Oguma N. (2002). Characteristic *S-N* properties of high-carbon-chromium-bearing steel under axial loading in long-life fatigue. Fatigue Fract. Eng. Mater. Struct..

[6] Shiozawa K., Lu L., Ishihara S. (2001). *S-N* curve characteristics and subsurface crack initiation behaviour in ultra-long life fatigue of a high carbon-chromium bearing steel. Fatigue Fract. Eng. Mater. Struct..

[7] Murakami Y., Yokoyama N.N., Nagata J. (2002). Mechanism of fatigue failure in ultralong life regime. Fatigue Fract. Eng. Mater. Struct..

[8] Grad P., Reuscher B., Brodvanski A., Kopnarski M., Kerscher E. (2012). Mechanism of fatigue crack initiation and propagation in the very high cycle fatigue regime of high-strength steels. Scr. Mater..

[9] Nakamura T., Oguma H., Shinohara Y. (2010). The effect of vacuum-like environment inside sub-surface fatigue crack on the formation of ODA fracture surface in high strength steel. Procedia Eng..

[10] Hong Y., Liu X., Lei Z., Sun C. (2016). The formation mechanism of characteristic region at crack initiation for very-high-cycle fatigue of high-strength steels. Int. J. Fatigue.

[11] Wang Q.Y., Bathias C., Kawagoishi N., Chen Q. (2002). Effect of inclusion on subsurface crack initiation and gigacycle fatigue strength. Int. J. Fatigue.

[12] Stanzl-Tschegg S. (1999). Fracture mechanisms and fracture mechanics at ultrasonic frequencies. Fatigue Fract. Eng. Mater. Struct..

[13] Stanzl-Tschegg S., Schönbauer B. (2010). Near-threshold fatigue crack propagation and internal cracks in steel. Procedia Eng..

[14] Murakami Y., Miller K. (2010). What is damage? A view point from the observation of low cycle fatigue process. Int. J. Fatigue.

[15] Tanaka K., Akiniwa Y. (2002). Fatigue crack propagation behavior derived from *S-N* data in very high cycle regime. Fatigue Fract. Eng. Mater. Struct..

[16] Akiniwa Y., Miyamoto N., Tsuru H., Tanaka K. (2006). Notch effect on fatigue strength reduction of bearing steel in the very high cycle regime. Int. J. Fatigue.

[17] Peterson R.E. (1974). Stress Concentration Factors.

[18] Murakami Y., Beretta S. (1999). Small defects and inhomgenities in fatigue strength: Experiments, models and statistical implications. Extremes.

[19] Pippan R., Tabernig B., Gach E., Riemelmoser F. (2002). Non-propagation conditions for fatigue cracks and fatigue in the very high-cycle regime. Fatigue Fract. Eng. Mater. Struct..

[20] Hertzberg R.W., Pecorini T.J. (1993). An examination of load shedding during fatigue fracture. Int. J. Fatigue.

[21] Li W., Deng H.L., Sun Z.D., Zhang Z.Y., Lu L.T., Sakai T. (2015). Subsurface inclusion-induced crack nucleation and growth behaviors of high strength steels under very High cycle fatigue: Characterization and microstructure-based modeling. Mater. Sci. Eng. A.

[22] Murakami Y. (2002). Metal Fatigue: Effects of Small Defects and Nonmetallic Inclusions.

